# Transport of Streptococcus pneumoniae Capsular Polysaccharide in MHC Class II Tubules

**DOI:** 10.1371/journal.ppat.0030032

**Published:** 2007-03-16

**Authors:** Tom Li Stephen, Mario Fabri, Laura Groneck, Till A Röhn, Helena Hafke, Nirmal Robinson, Jens Rietdorf, David Schrama, Jürgen C Becker, Georg Plum, Martin Krönke, Harald Kropshofer, Wiltrud M Kalka-Moll

**Affiliations:** 1 Institute for Medical Microbiology, Immunology and Hygiene, Medical Center, University of Cologne, Cologne, Germany; 2 Pharmaceutical Research, F. Hoffmann-La Roche AG, Basel, Switzerland; 3 Advanced Light Microscopy Facility, European Molecular Biology Laboratories, Heidelberg, Germany; 4 Department of Dermatology, Julius-Maximilians-University of Wuerzburg, Wuerzburg, Germany; National Institutes of Health, United States of America

## Abstract

Bacterial capsular polysaccharides are virulence factors and are considered T cell–independent antigens. However, the capsular polysaccharide Sp1 from Streptococcus pneumoniae serotype 1 has been shown to activate CD4^+^ T cells in a major histocompatibility complex (MHC) class II–dependent manner. The mechanism of carbohydrate presentation to CD4^+^ T cells is unknown. We show in live murine dendritic cells (DCs) that Sp1 translocates from lysosomal compartments to the plasma membrane in MHCII-positive tubules. Sp1 cell surface presentation results in reduction of self-peptide presentation without alteration of the MHCII self peptide repertoire. In DM-deficient mice, retrograde transport of Sp1/MHCII complexes resulting in T cell–dependent immune responses to the polysaccharide in vitro and in vivo is significantly reduced. The results demonstrate the capacity of a bacterial capsular polysaccharide antigen to use DC tubules as a vehicle for its transport as an MHCII/saccharide complex to the cell surface for the induction of T cell activation. Furthermore, retrograde transport requires the functional role of DM in self peptide–carbohydrate exchange. These observations open new opportunities for the design of vaccines against microbial encapsulated pathogens.

## Author Summary

Microorganisms are comprised of proteins, carbohydrates, lipids, and nucleic acids. Current immunologic paradigms state that activation of T lymphocytes required for humoral and cellular immune responses resulting in immunologic memory to the pathogens is solely brought about by proteinaceous antigens, processed and degraded to small peptides, loaded onto major histocompatibility complex (MHC) molecules, and transported as MHC/peptide complexes to the cell surface, where the MHC/peptide complex is recognized by the T cell antigen receptor. The findings of the present study elucidate the mechanism of MHC class II (MHCII)–dependent presentation of the bacterial capsular polysaccharide of Streptococcus pneumoniae serotype 1 (Sp1) that results in effective T cell activation. Sp1 is transported in MHCII-positive tubules from lysosomal compartments to the plasma membrane for presentation. In the absence of the DM molecule, known as an editor and catalyst of self and foreign peptide exchange, retrograde transport of carbohydrate/MHCII complexes resulting in dendritic cell engagement with T cells in vitro and T cell–dependent immune responses to the polysaccharide in vivo fail. The results suggest a fundamental shift in the immunologic paradigm, offering previously unrecognized opportunities for the design of new classes of vaccines against infectious diseases.

## Introduction

The immune response to polysaccharide antigens is considered T cell–independent [1]. However, emerging evidence suggests that bacterial polysaccharides from *Streptococcus pneumoniae, Bacteroides fragilis,* and Staphylococcus aureus activate CD4^+^ T cells in vivo and in vitro due to their zwitterionic charge motif within each repeating unit [2,3]. Nuclear magnetic resonance (NMR) structural studies of zwitterionic polysaccharides (ZPSs) such as the capsular polysaccharides PS A2 from B. fragilis and Sp1 from S. pneumoniae serotype 1 reveal the formation of extended right-handed helices with repeated 20 Å negatively charged grooves and positive charges located on the outer surfaces of the lateral boundaries [4,5]. A minimum molecular weight of ZPS >5 kDa and ≤17 kDa is required for the elucidation of antigenicity [6]. ZPSs induce CD4^+^ T cell activation in the presence of B cells, monocytes, and dendritic cells (DCs) [7] and have been demonstrated to correct systemic T cell deficiencies [8]. Animals lacking αβCD4^+^ T cells fail to develop abscesses in response to ZPS [9]. We and others have shown that T cell activation by the ZPS requires the costimulatory molecules B7–2, CD40, and the major histocompatibility complex (MHC) class II protein HLA-DR [7,10,11]. ZPSs locate in endosomal compartments and co-immunoprecipitate with HLA-DR [7,12]. These studies indicate similarities between ZPS and peptide antigen presentation to CD4^+^ T cells by antigen-presenting cells (APCs).

Antigen processing and presentation to CD4^+^ T cells by the MHCII endocytic pathway has been considered strictly limited to protein antigens [13]. A complex set of interlinked factors, including the local pH, is likely to influence the activity of the processing enzymes. The endosomal pH in APCs is regulated by proinflammatory and anti-inflammatory cytokines [14] and microbial products such as bacterial lipopolysaccharide (LPS) [15]. LPS triggers enhanced vacuolar proton ATPase function in immature DCs (iDCs), lower endosomal or lysosomal pH, and more efficient antigen processing and a rapid and transient boost of MHCII synthesis [15,16]. Another important event in antigen processing and presentation is the removal of class II invariant chain (CLIP) occupying the peptide binding groove by the MHCII homolog DM (HLA-DM in humans and H2-M in mice). DM further stabilizes the empty MHCII molecule and assists in peptide selection [17–19]. In the absence of DM, peptide editing fails, leading to the appearance of weakly bound peptides, including CLIP [20]. CLIP also qualifies as an endogenous regulator in DCs in priming of T helper cells by antagonizing the polarization towards the T_H_1 phenotype [21]. Recent studies show that LPS challenge induces tubules from lysosomes, which transport MHCII to the cell surface [22–24]. In the case of protein-loaded lysosomes, protein is transported in the MHCII tubules to the cell surface for presentation of the peptide, formation of biological interaction with T cells, and induction of T cell–dependent immune responses [24,25]. Although lysosomal compartments contain abundant glycosidases that act on sugar linkages, presumably with high specificity, they are considered the end stations for carbohydrates. MHCII tubules formed after LPS challenge in iDCs do not transport carbohydrates such as dextran from lysosomes to the cell membrane [24]. However, Cobb et al. recently showed that a nitric oxide–dependent processing mechanism of the ZPS PS A1 in early endosomes, resulting in the generation of low molecular weight, antigenic fragments [12]. This finding indicates that antigen processing is not limited to proteins only. Our recent observation that blockade of endo/lysosomal acidification inhibits carbohydrate-induced T cell activation [11] suggests that intravesicular acidic pH and DM activity [26] are required for polysaccharide binding to MHCII in lysosomal compartments and/or retrograde transport of carbohydrate/MHCII complexes.

Here, we report the mechanism of retrograde transport of the bacterial capsular polysaccharide Sp1 from S. pneumoniae in live cells and the essential role of DM in this process. Sp1 traffics through endosomal compartments to acidic lysosomes and is transported in MHCII-positive tubules for presentation to the cell surface and engagement of T cells. We show that the DM molecule is required for the retrograde transport and the cellular immune responses in vitro and in vivo. The data close a gap in our understanding of the new paradigm of MHCII presentation of bacterial carbohydrate antigens.

## Results

### Sp1 Endosomal Trafficking

**Figure 1:**
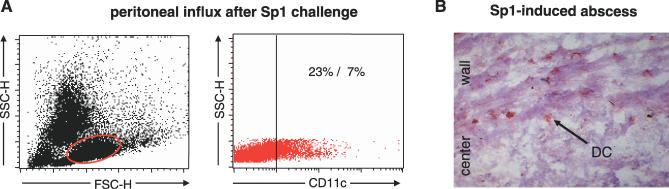
DCs in Intraabdominal Abscess Formation Induced by Sp1 (A) Twenty-four hours after intraperitoneal Sp1 challenge, cells of the peritoneal influx were stained with phycoerythrin (PE)–conjugated anti-CD11c mAb to stain DCs, and analyzed by flow cytometry. Numbers in the right quadrant of the right dot blot represent the percentage of CD11c-positive cells gated as indicated in the left dot blot and non-gated cells, respectively. FSC-H, forward scatter; SSC-H, sideward scatter. (B) Immunohistology of abscesses was performed with anti-CD11c mAb. One single CD11c-positive cell in the abscess wall is labeled with a black arrow.

**Figure 2:**
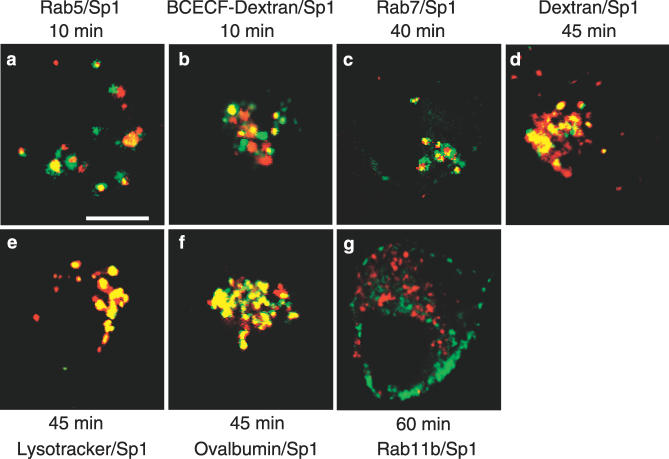
Intracellular Trafficking to Lysosomes of Sp1 in WT DCs (A) Merged fluorescence images of iDCs transfected with Rab5, a marker for early endosomes, and treated with Sp1-Alexa 594 (500 μg/ml) for 10 min. (B) Merged fluorescence images of iDCs incubated with BCECF-dextran (1 mg/ml), an indicator for early endosomal compartments with a pH of 6 to 7, for 45 min. iDCs were then incubated with Sp1-Alexa 594 (500 μg/ml) for 10 min. (C) Merged fluorescence images of iDCs transfected with Rab7, a marker for late endosomes, and incubated for 30 min after treatment with Sp1-Alexa 594 (500 μg/ml) for 10 min. (D) Merged fluorescence images of iDCs treated simultaneously for 45 min with Sp1-Alexa 594 (500 μg/ml) and dextran-Alexa Fluor 488 (1.5 mg/ml), a marker for late endosomes/lysosomes. (E) Merged fluorescence images of iDCs treated simultaneously for 45 min with Sp1-Alexa 488 (500 μg/ml) and LysoTracker Red DND-99 (50 nM), a marker for lysosomes with a pH of 3 to 5. (F) Merged fluorescence images of iDCs treated simultaneously for 45 min with Sp1-Alexa 594 (500 μg/ml) and ovalbumin-FITC (1 mg/ml). (G) Merged fluorescence images of iDCs transfected with Rab11b, a marker for recycling endosomes, and incubated for 1 h after treatment with Sp1-Alexa 594 (500 μg/ml) for 60 min.

Sp1 induces T cell activation in the presence of B cells, monocytes, and DCs [7]. In an experimental model of abscess formation, besides macrophages [27], CD11c-positive DCs play an important role. They migrate into the peritoneal cavity upon Sp1 challenge and are retrieved in the abscess capsule (Figure 1). Live cell imaging showed that in iDCs, part of Sp1 is internalized into early endosomes as indicated by partial co-localization with Rab5 and BCECF-dextran (dextran-2′,7′-bis-(2-carboxyethyl)-5-(and-6)-carboxyfluorescein) (Figure 2A and 2B). Co-localization of Sp1 with Rab7 and dextran, markers for late endosomes and lysosomes, and with LysoTracker, an acidotropic marker for lysosomes, demonstrated that Sp1-containing compartments fuse with late endosomes and lysosomes (Figure 2C–2E). Sp1 co-localized with ovalbumin, a conventional protein antigen processed and presented by the MHCII pathway (Figure 2F). In order to test whether Sp1 is internalized and represented by recycling receptors, we performed live cell imaging with Rab11b-EGFP fusion protein, a marker for recycling endosomal compartments. Co-localization of Sp1 with Rab11b was not observed during an observation interval of 5 min to 24 h in the absence and presence of LPS treatment (Figure 2G). These results demonstrate that after internalization, Sp1 gains access to endocytic compartments where antigenic epitopes are loaded to MHCII molecules. As presentation of Sp1 by recycling receptors is largely excluded, the question arises whether and how Sp1 is transported to the cell surface.

### Presentation of Sp1 on the Cell Surface via Retrograde Transport in MHCII Tubules

**Figure 3:**
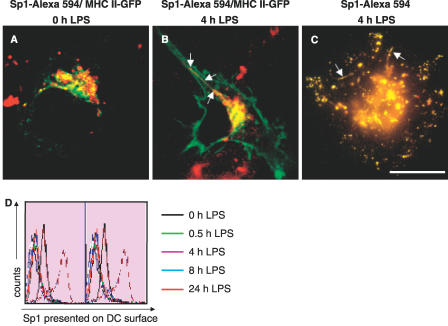
Export of Sp1 from Lysosomes to the Cell Membrane in MHCII-GFP–Positive Tubular Structures in Live DCs (A) iDCs transfected with MHCII-GFP were treated for 30 min with Sp1-Alexa 594 (500 μg/ml) and then analyzed by live cell imaging using confocal microscopy. In merged fluorescence images, Sp1 co-localizes extensively with MHCII in lysosomes. No MHCII is found on the cell surface. (B) iDCs transfected with MHCII-GFP were pre-incubated for 30 min with Sp1-Alexa 594 (500 μg/ml) and then treated with LPS for 4 h. Merged fluorescence live cell images of confocal microscopy demonstrate that Sp1/MHCII-positive tubular structures emanate from lysosomes in a perinuclear region and transit to the cell membrane. A single translocation Sp1/MHCII-positive tubule is labeled with white arrows (see Video S1). (C) iDCs previously transfected with MHCII-GFP and pre-incubated for 30 min with Sp1-Alexa 594 (500 μg/ml) were stimulated for 4 h with LPS. Combined TIR-FM/EPI microscopy reveals exit of Sp1-containing tubules from lysosomes (red) and their association with the plasma membrane (bright yellow). Tubules are labeled with white arrows (see Video S2). Scale bar, 10 μm. (D) After treatment with Sp1-biotin for 30 min, iDCs were incubated for various time intervals with LPS, and their cell surface was stained with FITC-conjugated streptavidin. Presentation of Sp1-biotin on the DC surface was quantified by flow cytometry.

Lysosomes constitute the terminal compartment of the endocytic pathway where exogenous components are generally degraded. Recent studies with green fluorescent protein (GFP)–tagged MHCII have shown that after LPS stimulation of iDCs, MHCII molecules are transported via tubules that originate from lysosomes to the plasma membrane [22–24]. We transfected iDCs of C57BL/6 wild-type (WT) mice with MHCII-GFP (I-Eα-EGFP) to investigate the presentation mechanism of Sp1. Flow cytometry analysis revealed that surface expression of assembled I-A and I-E molecules in transfected DCs was similar to I-A surface expression in non-transfected cells (Figure S1). Thus, MHCII-GFP was fully functional and appeared to exhibit the same general pattern of intracellular transport as endogenous MHCII in iDCs and mature DCs (mDCs). In iDCs, MHCII-GFP co-localized extensively with Sp1-Alexa 594 in lysosomes (Figure 3A). No MHCII-GFP was found on the cell surface. Within 4 h after LPS stimulation, numerous extensive tubules extended from the perinuclear area, which were intensely labeled for both MHCII and Sp1 (Figure 3B; see also Video S1). All tubules were yellow, indicating that Sp1 is exclusively transported in MHCII-positive tubules.

**Figure 4:**
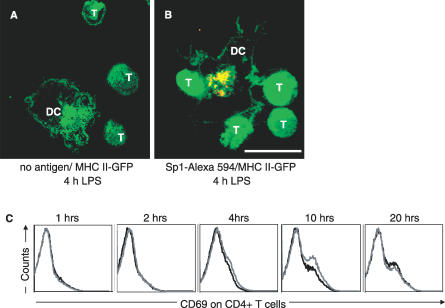
Interaction of DCs with Naïve CD4^+^ T Cells iDCs expressing MHCII-GFP and CFSE-labeled CD4^+^ T cells were used for live cell imaging of DC/T-cell immune interactions. Live fluorescent confocal microscopy imaging was performed at 4 h of LPS treatment. CD4^+^ T cells are indicated by a “T”. (A) DCs left untreated. (B) iDCs were pulsed for 30 min with Sp1 (500 μg/ml) and then treated with LPS. Scale bar, 10 μm. (C) iDCs were treated with Sp1 (100 μg/ml) for 45 min or left untreated before incubation with naïve CD4^+^ T cells in the presence of LPS (100 ng/ml). CD69 surface expression on CD4^+^ T cells was analyzed at indicated time points. Black line, non-Sp1-treated DCs; gray line, Sp1-treated DCs.

The observation that lysosomes can form dynamic and motile MHCII/Sp1-containing tubules does not prove that these structures mediate the transfer of MHCII/Sp1 complexes to the cell membrane. To determine directly whether these tubules not only move to the periphery but also actually reach the plasma membrane, we imaged Sp1 transport in DCs using combined epifluorescence (EPI) and total internal reflectance fluorescence microscopy (TIR-FM) [28]. We observed Sp1-Alexa 594–containing tubules exiting lysosomes and associating with the plasma membrane (Figure 3C; see also Video S2). With time, as the tubule approached the membrane, the red-colored EPI signal decreased while the bright yellow TIR-FM signal increased. After 4 h of LPS stimulation of Sp1-biotin–treated DCs, we detected significant amounts of Sp1 on the APC surface by fluorescence-activated cell sorting (FACS) analysis (Figure 3D), demonstrating that MHCII/Sp1 fuses with the cell membrane for cell surface presentation of Sp1. Concomitantly, Sp1-treated DCs induced engagement with CD4^+^ T cells, while LPS stimulation of DCs in the absence of Sp1 did not induce conjugate formation with T cells (Figure 4A and 4B). Analysis of CD69, the early activation marker, showed that although the majority of naïve CD4^+^ T cells form transient interactions with Sp1-pulsed DCs, a maximum 8% of naïve T cells are activated by Sp1-treated DCs at 10 h of incubation (Figure 4C), indicating proper cell surface T cell stimulatory function of MHCII/Sp1 complexes.

### Co-Presentation of Sp1, CLIP, and Self Peptides

**Figure 5:**
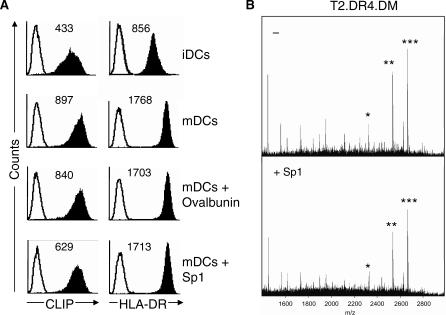
Effect of Sp1 on Self-Peptide Presentation (A) Flow cytometry analysis of iDCs, mDCs, ovalbumin (200 μg/ml)–treated, and Sp1 (200 μg/ml)–treated mDCs stained for CLIP and HLA-DR. Solid lines indicate the isotype-stained controls. The MFI is indicated in the upper corner. (B) MALDI-MS analysis of self peptides derived from T2.DR4.DM transfectants, without and with Sp1 treatment (200 μg/ml). MHCII HLA-DR molecules were precipitated with mAb L243. * indicates CLIP(81–102) m/z = 2333.4; **, CLIP(81–103) m/z = 2543.4; and ***, CLIP(81–104) m/z = 2674.5.

The proposed functions of CLIP are that of a precursor peptide to be exchanged for foreign antigenic peptides, and of a regulator in priming T_H_ cells by antagonizing the polarization towards the T_H_1 phenotype [21]. It was shown that regardless of the presence and type of protein antigen provided to mDCs and loaded onto MHCII, the number of surface CLIP/MHCII complexes remained unchanged [21]. Here, in contrast to ovalbumin as a control antigen, the incubation of maturing DCs with Sp1 resulted in a 57% decrease in Δ-CLIP surface expression (Figure 5A), whereas HLA-DR expression remained unaltered. The ratio of the mean fluorescence intensities (MFIs) of CLIP/MHCII for iDCs, mDCs, and ovalbumin-treated DCs was 0.5, and 0.3 for Sp1-treated DCs. This observation suggests that CLIP is displaced when Sp1 is present and that reduced CLIP surface presentation modulates Sp1-mediated T cell immune responses. To investigate whether Sp1 treatment also affects presentation of self peptides other than CLIP, we performed matrix-assisted laser desorption and ionization mass spectrometry (MALDI-MS) of MHCII precipitates from T2.DR4.DM transfectants. The composition of the self-peptide repertoire with CLIP as the major representative remained unaltered when we compared MALDI-MS spectra obtained in the absence and presence of Sp1 (Figure 5B). In summary, Sp1 provided to DCs and loaded onto MHCII leads to a reduction in the number of surface self-peptide/MHCII complexes with CLIP/MHCII as the principal subset.

### Requirement of DM for T Cell–Dependent Immune Responses to Sp1 In Vivo

**Figure 6:**
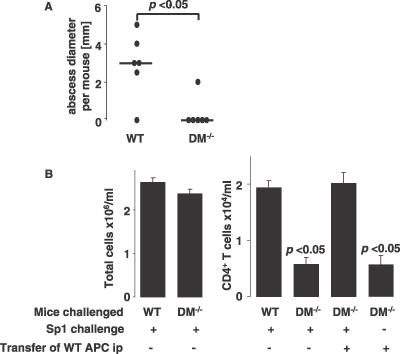
DM Dependency of Sp1-Mediated T Cell–Dependent Immune Responses (A) WT and DM^−/−^ were challenged with Sp1 and intraperitoneal abscess formation was measured after 6 d. (B) The total cellular influx (left panel) and the influx of CD4^+^ T cells (right panel) into the peritoneal cavity was evaluated in WT and of DM^−/−^ mice 24 h after Sp1 challenge and/or adoptive transfer.

Sp1 is a highly charged molecule and might be exchanged for peptides in an antigen site due to its stronger electrostatic forces. However, it is possible that DM as a catalyzer of peptide exchange and editor of peptide/MHCII binding might also be required for carbohydrate/peptide exchange. To assess the catalytic activity of DM, we first investigated whether DM is required for T cell–dependent immune responses to Sp1 in vivo. In an experimental model for abscess formation, unlike WT mice, animals lacking DM are not able to form abscesses in response to Sp1 (Figure 6A). Twenty-four hours before challenge, CD4^+^ T cells from WT mice were adoptively transferred to DM^−/−^ mice per intravenous route to compensate for the 3- to 4-fold reduction of mature CD4^+^ T lymphocytes and for the diminished T cell repertoire selection of DM^−/−^ mice [29]. Analysis of the cells migrating into the peritoneal cavity 24 h after polysaccharide challenge showed that the total number of cells did not differ in WT and DM^−/−^ mice (Figure 6B, left panel). In both groups, about 40% of influx cells were macrophages (not shown). In contrast to WT mice, the peritoneal influx of CD4^+^ T cells was significantly reduced in DM^−/−^ mice (Figure 6B, right panel). Adoptive intraperitoneal transfer of Sp1-pulsed APCs from WT mice fully reconstituted the CD4^+^ influx in DM^−/−^ mice. Analysis of the peritoneal lavage did not reveal a CD4^+^ T cell influx to the APC transfer alone. These findings indicate that the CD4^+^ T cell influx in response to Sp1 depends on DM expression in peritoneal APCs.

### Requirement of DM for Sp1 Retrograde Transport

**Figure 7:**
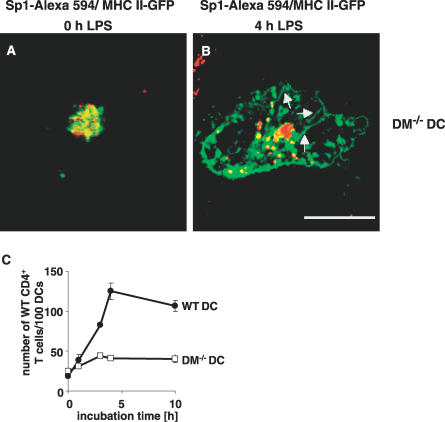
DM Dependency of Sp1 Retrograde Transport in MHCII-Positive Tubules (A) DM^−/−^ iDCs transfected with MHCII-GFP were treated for 30 min with Sp1-Alexa 594 (500 μg/ml) before live cell imaging by confocal microscopy. Merged fluorescence images of DM^−/−^ iDCs reveal co-localization of Sp1 with MHCII-GFP in lysosomes. No MHCII is found on the cell surface. (B) DM^−/−^ iDCs transfected with MHCII-GFP were pre-incubated with Sp1-Alexa 594 (500 μg/ml) for 30 min and then treated with LPS for 4 h. Merged fluorescence live cell images of confocal microscopy demonstrate that DCs form MHCII-GFP–positive tubules by 4 h of stimulation with LPS that extend from the perinuclear area to the cell surface (arrows) while Sp1-Alexa 594 remains lysosomal (right panel). Scale bar, 10 μm. (C) DCs from WT or DM^−/−^ mice expressing MHCII-GFP were stimulated with Sp1-Alexa 594 (500 μg/ml) for 30 min and LPS for various time intervals. Immune interactions with CFSE-labeled WT T cells were analyzed by live cell fluorescent microscopy imaging (see also Figure 2E and 2F).

We investigated the role of DM in the retrograde transport of Sp1 in DCs and in the initiation of T cell–dependent immune responses in vitro. We transfected iDCs of DM^−/−^ mice of the H-2b haplotype with I-Eα-EGFP. The dependency of I-A molecules on DM to function in antigen presentation has been characterized extensively in the DM^−/−^ mouse strain used [29–31]. It also has been shown that I-E is dependent on DM for peptide loading, as evidenced by the abundance of CLIP occupying the MHCII groove in DM^−/−^ mice [32]. Flow cytometry analysis revealed that after LPS stimulation of DM^−/−^ iDCs, assembled I-E and I-A molecules appeared at the cell surface with similar quantities and kinetics as in WT DCs (Figure S2). In DM^−/−^ iDCs, MHCII co-localized extensively with Sp1 in lysosomes (Figure 7A). Within 4 h of stimulation with LPS, tubules extended from the perinuclear area, which were intensely labeled for MHCII-GFP and were devoid of Sp1-Alexa 594 (Figure 7B). At this time point and within the next 6 h, all tubules were green, indicating that Sp1-Alexa 594 is not transported in tubules with MHCII-GFP in DCs lacking DM. To provide functional evidence for the requirement of DM for Sp1 presentation in DCs, we examined the effect of the absence of DM on APC/T-cell engagement in vitro. iDCs from DM^−/−^ and WT mice were pulsed with Sp1-Alexa 594 in the presence or absence of LPS for different time intervals. Pulsed DCs were incubated with carboxyfluorescein succinimidyl ester (CFSE)–labeled CD4^+^ T cells from WT mice and examined for DC/T-cell conjugate formation by fluorescent microscopy. After the addition of LPS, WT DCs showed a significant increase of APC/T-cell conjugates, which peaked at 4 h to 10 h (Figure 7C). In contrast to WT DCs, DM^−/−^ DCs pulsed with Sp1-Alexa 594 did not induce significant conjugate formation with CD4^+^ T cells.

## Discussion

The new paradigm of MHCII-restricted presentation of carbohydrates leaves open obvious questions regarding the precise mechanism of bacterial capsular carbohydrate interactions with MHCII molecules. Here we provide evidence that internalization of polysaccharides is followed by intracellular transport and presentation on the cell surface by newly synthesized MHCII molecules. We show that in DCs, Sp1 migrates in tubules as carbohydrate/MHCII complexes to the cell surface to induce T cell–dependent immune responses in vitro and in vivo. Sp1/MHCII retrograde transport requires the editor protein DM.

Intracellular tracking of Sp1 reveals partial co-localization with BCECF-dextran and Rab5, markers for early endosomes, that might reflect different pathways for Sp1-containing pinocytic and endocytic vesicles [33] or an intermediate status during fast maturation of Sp1-containing vesicles into late endosomes and lysosomes. In early endosomes, Sp1 could be subjected to oxidation by free radicals as was shown for PS A1 [12]. There is an increasing acidification of Sp1-containing intracellular compartments. We previously demonstrated that Sp1-induced T cell activation depends on the acidic lysosomal pH and that Sp1 induces maturation of human monocyte-derived DCs [11]. Besides proteases, lysosomes also contain abundant glycosidases, such as fucosidases and galactosidases [34]. At a later stage of the endocytic pathway at an optimal acidic pH of maturing DCs [15], glycosidases may trim Sp1 to smaller molecular sizes, forming conformations that facilitate Sp1 anchoring and binding to MHCII and promote optimal generation of T cell epitopes.

In contrast to non-charged dextrans, Sp1 is transported from lysosomes to the cell surface in MHCII-positive tubules like conventional protein antigens HEL and Ova [24,25]. Confocal, EPI/TIR fluorescence microscopy, and FACS analyses demonstrate time-dependent retrograde transport and cell surface presentation of Sp1 on maturing DCs, indicating fusion of Sp1-carrying tubules with the plasma membrane for formation of an immunological synapse required for proper activation of T cells. Presentation of Sp1 on the DC surface results in conjugate formation with a considerable number of T cells from non-Sp1–primed naïve mice. Although mDCs are known to attract and cluster with naïve T cells [35], it is possible that T cells from non–germ-free animals that are colonized with the ubiquitous gut organism B. fragilis are primed by the ZPS from B. fragilis and cross-react with Sp1 [36]. Indeed, about 8% of the naïve CD4^+^ T cells become activated by Sp1-treated DCs. The drastically different immunogenic properties of Sp1 are brought about by specific biochemical characteristics by which Sp1 distinguishes itself from other carbohydrates such as dextrans. At an acidic lysosomal pH similar to the Sp1 isoelectric point of 3.5 (unpublished observation), an optimal equilibrium of positively charged free amino groups and negatively charged carboxyl groups is available and provides a large number of binding sites to associate with MHCII molecules. The high density of alternating opposite charges is exposed on the outmost surface of the molecule. Maximum binding would be achieved via abundant electrostatic interactions supplemented by the potential for numerous hydrogen bonds to hydrophilic hydroxyls and van der Waals interactions.

The proper balance of T_H_1 and T_H_2 immunologic responses is critical to maintain balance in the immune system's task to fight microbial antigens. It has been shown that increased representation of CLIP/MHCII complexes antagonize polarization of T cells towards the T_H_1 phenotype [21]. Here, we show reduction of CLIP cell surface presentation in mDCs possibly caused by antigenic exchange of CLIP with Sp1 and/or modulation of MHCII/self-peptide retrograde transport by Sp1. Inhibition of presentation of CLIP/MHCII in Sp1-treated mDCs might contribute to the establishment of a T_H_1/T_H_2 balance towards the T_H_1 phenotype as has been described for the ZPS of the symbiotic intestinal bacteria B. fragilis [8]. It might also be responsible for low Sp1-specific antibody production (unpublished data) and might modulate the immune response to ZPS during abscess formation and adhesion.

Beyond the functional role of DM in peptide exchange, our data suggest an extension to antigenic exchange with carbohydrates. Three functions have been described for DM: 1) to catalyze the removal of CLIP or non-CLIP peptides and their exchange by heterogeneous peptides [17–19]; 2) to serve as a molecular chaperone, preventing non-specific aggregation of the temporarily empty αβ dimers following CLIP release [37]; and 3) to function as a peptide editor, positively selecting peptides that can stably bind to a particular class II allele [18,38]. Besides facilitating Sp1 binding through catalytic release of CLIP and other peptides, DM might select those Sp1 length variants for binding that form the most stable complexes due to their optimal structural and electrostatic features. Although in DM-deficient DCs accumulation of Sp1 in endocytic compartments, retrograde transport, and surface expression of MHCII complexed with either CLIP or self peptides is normal, they are inefficient in transporting Sp1 from endocytic compartments to the cell surface and initiating conjugate formation with naïve CD4^+^ T cells.

So far, it is not possible to rule out internalization and presentation of ZPS by recycling MHCII or presentation similar to superantigens [7,12]. Recycling MHCII binds to peptides in early endosomes and traffics between early endosomes and cell membrane. Indeed, Sp1 partially co-internalizes with transferrin (not shown), a marker for recycling receptor-mediated endocytosis. However, Sp1 is directed from early endosomal compartments to late endosomes and lysosomes, where it co-localizes with newly synthesized MHCII and does not locate in recycling endocytic Rab11b-positive compartments. Furthermore, the dependency of Sp1 biological activity on DM and on the retrograde tubular transport of MHCII clearly argues against a presentation mechanism similar to superantigens and by recycling MHCII molecules.

Taking the results together, we show that bacterial polysaccharide–induced APC/T-cell conjugate formation and T cell–dependent immune responses depend on retrograde transport via MHCII tubules and the functional role of DM.

## Materials and Methods

### Antigens.

S. pneumoniae type 1 capsular polysaccharide complex was obtained from the American Type Culture Collection (http://www.atcc.org) and further purified to obtain homogeneity as described previously [11]. High-resolution (500 MHz) proton NMR spectroscopy [5] revealed that Sp1 was free of contaminating protein and nucleic acids. Endotoxin was not detectable in Sp1 by the limulus test with sensitivity of <8 pg LPS/mg Sp1. As control antigens, ovalbumin–fluorescein isothiocyanate (FITC), ovalbumin–Alexa Fluor 594, dextran–Alexa Fluor 488, and dextran–Texas Red Molecular Probes were used (http://probes.invitrogen.com).

### Inhibitors and markers.

For intracellular tracking, BCECF-dextran, LysoTracker Red DND-99, dextran–Alexa Fluor 488, and ovalbumin-FITC were obtained from Molecular Probes.

### Labeling of ZPS.

Sp1 is a linear polymer of an average molecular size of 90 kDa corresponding to 167 trisaccharide repeating units with a respective molecular size of 537 Da. Each repeating unit of Sp1 contains one positively and two negatively charged groups with galacturonic acid (GalA, residues a and c) and 2-acetamido-4-amino-2,4,6-trideoxygalactose (Aat, residue b) with a sequence of →3)-α-D-GalA (a)-(1→3)-α-D-Aat (b)-(1→4)-α-D-GalA (c)-(1→ [5,39]. The adjacent hydroxyl groups on residue c (molecular weight 175) were oxidized by sodium m-periodate (Sigma, http://www.sigmaaldrich.com) treatment in molar ratios ranging from 1:0.1 to 1:0.5 for 90 min at room temperature in the dark to create highly reactive aldehyde functional groups [40]. The reaction was stopped by addition of ethylene glycol (Sigma). After gel filtration chromatography with a PD-10 column (Amersham, http://www.amersham.com), Sp1 was labeled by formation of covalent hydrazone linkages between aldehydes and EZ-Link Biotin-Hydrazide (Pierce, http://www.piercenet.com), Alexa Fluor 488 hydrazide, and Alexa Fluor 594 hydrazide (Molecular Probes) following the instructions of the manufacturer. After reduction of residual aldehydes of biotinylated Sp1 (Sp1-biotin), Alexa Fluor 488–labeled Sp1 (Sp1-Alexa 488), and Alexa Fluor 594–labeled Sp1 (Sp1-Alexa 594) by base treatment at pH 9.0 for 60 min, the glycoconjugate was separated from unbound labeling agents by three consecutive runs on PD-10 columns. The degree of biotinylation was determined with the ImmunoPure 2-(4′-hydroxyazobenzene benzoic acid) (HABA) and ImmunoPure Avidin (Pierce) reagents, following the instructions of the manufacturer. This method allows the calculation of mol biotin per mol Sp1 and number of biotin molecules per repeating units. Labeled Sp1 carried a biotin molecule on every 20th repeating unit (Sp1-biotin), which corresponds to one label per 11-kDa fragment by ^1^H NMR spectroscopy and showed the same chemical shifts as native Sp1 (Figure S3A and S3B). The additional signals obtained for Sp1-biotin originated from EZ-Link Biotin-Hydrazide (Figure S3B, upper spectrum). All mice challenged with Sp1-biotin developed intraabdominal abscesses to the same degree as native Sp1 (Figure S3C). Sp1 labeled with Alexa Fluor hydrazide 488 (Sp1-Alexa 488) or Alexa Fluor hydrazide 594 (Sp1-Alexa 594) preserved its intact structure and in vivo immune responses (not shown). These controls demonstrated that the biological activity of labeled Sp1 used in our studies is indistinguishable from that of unlabeled Sp1.

### NMR spectroscopy.

NMR spectra were obtained from a sample of 2 mg of purified Sp1, Sp1-biotin, or Sp1-Alexa 488, which was exchanged with ^2^H_2_O once and redissolved in 0.7 ml of ^2^H_2_O as described previously [5]. NMR experiments were performed on a Bruker DRX 500 instrument (Bruker, http://www.bruker.de) with a proton resonance frequency of 500.13 MHz. The ^1^H spectra were recorded at 80 °C in ^2^H_2_O using presaturation to suppress the water signal. Chemical shifts were referenced in relation to ^1^H^2^HO resonance at 4.36 ppm.

### Abscess induction studies and evaluation of the peritoneal influx.

Animal experiments were performed in accordance with the guidelines of German animal protection legislation (license number 50.203.2-K 16,3/02). In abscess induction studies, B6129SF2/J (WT) and H2-Dma*^tm1Luc^* (DM^−/−^) [29] obtained from Charles River Laboratories (http://www.criver.com) were injected intraperitoneally with Sp1 (100 μg of Sp1 in PBS mixed with sterile cecal content adjuvant [SCCA]; 1:1 v/v, 0.2 ml total volume) [9]. Then, 24 h before challenge, 2 × 10^7^ CD4^+^ T cells (>95% purity) from WT mice were adoptively transferred to DM^−/−^ mice per intravenous route. Six days after challenge, mice were macroscopically examined for the presence of abscesses within the peritoneal cavity by two double-blinded examiners. Abscesses were isolated and their diameter was measured.

The cellular influx into the peritoneal cavity was assessed at 24 h following challenge with Sp1. As in abscess induction studies, 2 × 10^7^ CD4^+^ T cells from C57BL/6 (WT) mice obtained from Charles River Laboratories were adoptively transferred to DM^−/−^ mice per intravenous route 24 h before challenge. WT mice were challenged intraperitoneally with Sp1. DM^−/−^ mice were either challenged intraperitoneally with Sp1, Sp1 plus 2 × 10^7^ WT APCs, or 2 × 10^7^ WT APCs alone. APCs were purified from the peritoneal lavage followed by CD4^+^ T cell depletion (<0.05% CD4^+^ T cells) of WT mice challenged 24 h before adoptive transfer. Mice underwent peritoneal lavage with 4 ml of ice-cold PBS. A total cell count was performed by trypan blue staining with a hemocytometer. Each sample was then analyzed by flow cytometry for different cell types. The absolute number of each respective cell type present was calculated by taking its respective frequency and multiplying it by the total number of cells per ml lavage obtained from each mouse. In each experiment, four to six mice per group were tested. The experiment was performed three times in an independent manner.

### Immunohistology.

Frozen sections of abscesses were fixed in cold acetone for 10 min followed by blocking of endogenous peroxidase with peroxidase blocking solution (DAKO, http://www.dako.com) for 10 min at room temperature. The CD11c antibody (N418, supernatant; 1:100 diluted) was then overlayed and the slides incubated in a humid chamber for 45 min. With TRIS washes between every step, a biotinylated link antibody (Becton Dickinson, http://www.bdbiosciences.com) was applied for 45 min followed by a streptavidin-alkaline phosphatase (DAKO) for 10 min. After another wash, the substrate (Vector NovaRed; Vector Laboratories, http://www.vectorlabs.com) was added and the slides were incubated in the dark for 20 min. After a TRIS wash, the slides were counter stained, mounted, and viewed using a Zeiss Axiophot microscope with photographic capabilities (http://www.zeiss.com).

### APCs and cell culture.

DCs were generated from mouse bone marrow by adapting a previously described method [41]. In brief, bone marrow cells from H2-Dma*^tm1Luc^* [29] (DM^−/−^) and C57BL/6 (WT) mice that have a mutation that abolishes production of the MHCII I-Eα chain [31,42] were cultured in RPMI supplemented with 5% FBS, 500–1,000 U recombinant mouse granulocyte/macrophage-colony stimulating factor (GM-CSF), 20 μg per ml gentamicin, and 50 μM 2-mercaptoethanol (DC medium). DC medium was exchanged in two-day intervals. DCs were isolated by magnetic cell sorting with a CD11c-specific monoclonal antibody (mAb) (Miltenyi Biotec, http://www.miltenyibiotec.com). CD11c-positive iDCs were imaged on days 4 and 5 of culture. mDCs were generated by adding LPS O26:B6 (100 ng/ml) (Sigma) to disaggregated and replated cultures.

Infection of proliferating precursors with retrovirus containing I-Eα-EGFP was performed on day 2 by adapting a method described previously by Chow and coworkers [24]. I-Eα-EGFP, kindly provided by I. Mellman, was cloned into LZRS-pBMN using EcoRI sites. This viral vector was transfected into ΦNX-ecotropic cells using calcium chloride. Virus was collected in DC medium for 24 h, supplemented with polybrene and HEPES, and added to the DC culture for infection. Cells were spun at 32 °C, 2,500 rpm for 2 h. Virus was removed and fresh medium added. Expression was assayed 48 h after infection.

Localization of Sp1 in recycling, early, and late endosomes was performed by adenoviral infection of live dendritic cells with Rab11b-EGFP, Rab5-GFP, and Rab7-GFP–containing mammalian expression plasmids [43]. DNA fragments containing Rab fusion protein constructs and CMV promoter were amplified in the double digested promoter-less transfer vector pEntry148AU-MCS. For packaging into adenovirus particles, constructs were recombined into pAd/PL-DEST vector (Invitrogen, http://www.invitrogen.com). Adenoviral stocks were produced in 293A cells after transfection with plasmid DNA and Lipofectamine. Infection of DCs with Rab5-EGFP, Rab7-EGFP, and Rab11b-EGFP was performed on day 4. iDCs were spun at 2,500 rpm at 37 °C for 120 min in virus-containing medium supplemented with 10 mM HEPES. After replacement with DC medium, expression was checked by fluorescence microscopy after 24 h. Transfection efficiency was 50% to 70%.

Human DCs were differentiated from peripheral blood mononuclear cells as described [44]. Monocytes were isolated from peripheral blood mononuclear cells by positive selection by anti-CD14 magnetic beads (Miltenyi Biotec) and cultured in complete RPMI medium containing 50 ng/ml GM-CSF and 3 ng/ml IL-4. Maturation was induced at day 4 by the addition of LPS from *Salmonella abortus equi* (1 μg/ml) (Sigma).

### Flow cytometry.

Staining of surface molecules was performed using PE- or FITC-conjugated anti-CD4 (clone L3T4), anti-CD11c (clone Hl3), anti-CD69 (clone HI.2F3), anti-I-Eαβ (clone 14.4.4S), anti-I-Aβ (clone AF6–120.1), anti-HLA-DR/CLIP complex (clone Cer.CLIP), and anti-HLA-DR (clone L243) (BD Pharmingen, http://www.bdbiosciences.com). For Sp1 surface presentation studies, iDCs were incubated for 30 min at 37 °C without or with Sp1-biotin (200 μg/ml), washed, and incubated for 30 min to 8 h at 37 °C in LPS-containing medium (100 ng/ml) before staining with streptavidin-FITC (Sigma) for 30 min and washing. Cells prepared for flow cytometry were analyzed—after gating for viable cells by forward and side scatter and by propidium iodide staining—by FACScan (Becton Dickinson) using CELLQuest software (Becton Dickinson). The results were expressed as MFI, or as percentage (%) of fluorescence-labeled APCs of the whole APC population.

### Live fluorescent microscopy.

To investigate intracellular trafficking of Sp1, live cell imaging was performed. Cells were plated for 30 min on poly-d-lysine–pre-coated number 1.5 coverslips attached to 35-mm dishes (MatTek, http://www.mattek.com), and fresh medium was added. To study mechanisms of internalization of Sp1, cells were incubated with competitors or chemical inhibitors for 30 min at 4 °C or 37 °C before Sp1 treatment. To monitor Sp1, APCs were loaded with markers for cellular compartments before or at the same time point of Sp1 addition. Cells were washed before and after Sp1 treatment three times in ice-cold medium. Inverted fluorescent microscopy was performed on an Olympus IX81 microscope (http://www.olympus-europe.com/microscopes/index.htm). Temperature control at 37 °C was achieved with a heating dish. Acquisition was performed using AnalySIS Imaging System software (Olympus, http://www.olympus.de). Confocal microscopy was done on a PerkinElmer UltraView LCI spinning disc system (http://las.perkinelmer.com) equipped with a suitable multi-band beamsplitter and a MellesGriot Omnichrome 643-RYB-A02 ArKr gas laser (http://www.mellesgriot.com) providing 488-nm and 568-nm lines for excitation. A Nikon Plan Fluor ×100 1.3NA oil immersion objective (http://www.nikon.com) and 525/50 and 607/45 emitter filters were used for GFP, FITC, Alexa 488, and Alexa 594, and Texas Red stains, respectively.

Multi-color TIR-FM and EPI was performed on an Olympus Biosystems Cell-R system equipped with a stabilized Xenon arc lamp and dual coupling for Coherent Sapphire 488–20 and Compass 250M-50 diode lasers (http://www.coherent.com) providing 488 nm and 532 nm excitation light, respectively. On confocal microscopy, EPI, and TIR-FM systems, environmental condition was controlled by a custom incubator (EMBL GP 168) that provides a 37 °C and 5% CO2 atmosphere. Images were exported to TIFF images, processed using Adobe Photoshop version 6.0 (http://www.adobe.com), and converted into QuickTime movies using Graphic Converter version 3.8 (Softguide, http://www.softguide.de).

### APC/T-cell engagement.

Investigation of DC/T-cell conjugate formation was performed as previously described [45]. In brief, DCs from C57BL/6 WT and DM^−/−^ mice were loaded with Sp1-Alexa 594 for 30 min or left untreated and washed. DCs (0.5 × 10^5^/ml) were then treated with LPS (100 ng/ml) for different time intervals, washed, and mixed with CFSE-labeled CD4^+^ T cells (1.5 × 10^5^) from C57BL/6 WT mice. Cells were centrifuged for 5 min at 50*g* to increase cell interactions, and incubated at 37 °C for 20 min. The cells were gently transferred to poly-d-lysine–pre-coated number dishes. After incubation at 37 °C for 30 min, T-cell–DC conjugates were subjected to imaging by fluorescent microscopy. CFSE-labeled T cells were distinguished from GFP-labeled DCs by morphology. Three independent experiments were performed and the number of CFSE-positive CD4^+^ T cells interacting with 100 Sp1-Alexa 594–positive DC was counted in a blinded manner as previously described [22].

For the investigation of WT CD4^+^ T cell activation induced by Sp1-treated DCs, the same protocol as for DC/T-cell conjugate formation was applied with some modifications. DCs were treated with 100 μg/ml Sp1 for 45 min or left non-treated. Analysis of the expression of CD69, the early activation marker on CD4^+^ T cells, was performed by flow cytometry at different time points after addition of T cells to DCs.

### Mass spectrometry.

T2.DR4.DM transfectants, expressing the MHCII molecules HLA-DR4 and DM, respectively, were maintained in RPMI 1640 supplemented with 10% FCS. T2 is a BxT cell hybrid with a large deletion in the MHCII locus and does not express endogenous MHCII proteins. Cells at a density of 6 × 10^5^ cells/ml were treated with Sp1 (200 ug/ml) for 20 h, washed with PBS, and lysed (6 × 10^6^/ml) at 4 °C in lysis buffer of 20 mM and 5 mM MgCl containing 1% Triton X-100 and protease inhibitors. The cells were precipitated with mAb L243 (recognizing antigen/HLA-DR complexes) conjugated to sepharose beads. Peptides were eluted with 0.1% trifluor-acetic acid. MALDI-MS analysis was done as described [21] on a Reflex III mass spectrometer (Bruker).

### Statistical analysis.

Comparison of groups with regard to abscess formation was made by chi-square analysis. Results of the various groups in peritoneal cellular influx and APC/T-cell engagement assays were compared by Student's *t* test.

## Supporting Information

Figure S1 Biological Activity of C57BL/6 DCs Transfected with MHCII Protein I-Eα-EGFP CD11c-positive DCs non-transduced or transduced with MHCII protein I-Eα-EGFP were treated in medium alone or with LPS for 4 h and 10 h. Surface expression of the MHCII proteins I-A and I-E was determined by flow cytometry.
(56 KB PPT)

Figure S2 Biological Activity of DM^−/−^ DCs Transfected with MHCII Protein I-Eα-EGFP CD11c-positive DM^−/−^ DCs non-transduced or transduced with MHCII protein I-Eα-EGFP left untreated or were treated with LPS for 4 h and 10 h. Surface expression of the MHCII proteins I-A and I-E was determined by flow cytometry.
(49 KB PPT)

Figure S3 Structure and Functional Activity of Biotin-Labeled Sp1 (A) ^1^H NMR spectra of native Sp1, (B) EZ-Link Biotin-Hydrazide (upper spectrum), and Sp1-biotin (lower spectrum) were recorded at 70 °C. (C) Sp1 and Sp1-biotin were tested in an animal model for intraabdominal abscess formation. Negative control: Challenge with SCCA alone. Each dot represents the total abscess diameter per mouse. The bar illustrates the median of total abscess diameters per group.
(1.0 MB PPT)

Video S1 Export of Sp1 from Lysosomes to the Cell Periphery in MHCII-GFP–Positive Tubular Structures in Live DCs Confocal imaging shows a C57BL/6 DC expressing MHCII-GFP (green), loaded with Sp1-Alexa 594 (red) for 30 min, and exposed to LPS for 4 h. MHCII-GFP partially localizes in late endosomes and lysosomes and on the cell surface. Sp1-Alexa 594 accumulates intracellularly in lysosomal compartments and is transported in MHCII-GFP tubules extending from the perinuclear lysosomal compartments to the cell surface. Time interval: 0.25 s/frame. Time duration: 1 min.
(118 KB MOV)

Video S2 Export of Sp1 from Lysosomes for Presentation on the Cell Membrane in MHCII-GFP–Positive Tubular Structures in Live DCs Combined TIR-FM and EPI live cell imaging shows a C57BL/6 DC expressing MHCII-GFP, loaded with Sp1-Alexa 594 for 30 min, and exposed to LPS for 4 h. Red-colored tubules show Sp1-containing compartments exiting lysosomes, while the bright yellow–colored structures represent Sp1-containing tubules reaching and associating with the plasma membrane. Some tubules are indicated by white arrows. Time interval: 0.2 s/frame. Time duration: 2.5 min.
(584 KB MOV)

## Abbreviations

APC: antigen-presenting cell
CLIP: class II invariant chain
CFSE: carboxyfluorescein succinimidyl ester
DC: dendritic cell
EPI: epifluorescence
FITC: fluorescein isothiocyanate
GFP: green fluorescent protein
iDC: immature dendritic cell
LPS: lipopolysaccharide
MALDI-MS: matrix-assisted laser desorption and ionization mass spectrometry
mAb: monoclonal antibody
mDC: mature dendritic cell
MHC: major histocompatibility complex
NMR: nuclear magnetic resonance
Sp1: *Streptococcus pneumoniae* serotype 1 capsular polysaccharide
TIR-FM: total internal reflectance fluorescence microscopy
WT: wild-type
ZPS: zwitterionic polysaccharide
